# Ionizing radiation downregulates estradiol synthesis via endoplasmic reticulum stress and inhibits the proliferation of estrogen receptor-positive breast cancer cells

**DOI:** 10.1038/s41419-021-04328-w

**Published:** 2021-10-29

**Authors:** Pengfei Yang, Xiu Feng, Jin Li, Tianyi Zhang, Chengyan Sheng, Liying Zhang, Junrui Hua, Wenjun Wei, Nan Ding, Jinpeng He, Yanan Zhang, Jufang Wang, Heng Zhou

**Affiliations:** 1 Key Laboratory of Space Radiobiology of Gansu Province and Key Laboratory of Heavy Ion Radiation Biology and Medicine, Institute of Modern Physics, Chinese Academy of Sciences, Lanzhou, China; 2 University of Chinese Academy of Sciences, Beijing, China; 3 School of Nuclear Science and Technology, Lanzhou University, Lanzhou, China; 4 Gansu University of Chinese Medicine, Lanzhou, China

**Keywords:** Breast cancer, Chaperone-mediated autophagy, Cell death

## Abstract

Breast cancer is a major threat to women’s health and estrogen receptor-positive (ER^+^) breast cancer exhibits the highest incidence among these cancers. As the primary estrogen, estradiol strongly promotes cellular proliferation and radiotherapy, as a standard treatment, exerts an excellent therapeutic effect on ER^+^ breast cancer. Therefore, we herein wished to explore the mechanism(s) underlying the inhibitory effects of radiation on the proliferation of ER^+^ breast cancer cells. We used the ER^+^ breast cancer cell lines MCF7 and T47D, and their complementary tamoxifen-resistant cell lines in our study. The aforementioned cells were irradiated at different doses of X-rays with or without exogenous estradiol. CCK8 and clone-formation assays were used to detect cellular proliferation, enzyme-linked immunosorbent assay (ELISA) to determine estradiol secretion, western immunoblotting analysis and quantitative real-time PCR to evaluate the expression of proteins, and immunofluorescence to track endoplasmic reticulum stress-related processes. Finally, BALB/C tumor-bearing nude mice were irradiated with X-rays to explore the protein expression in tumors using immunohistochemistry. We found that ionizing radiation significantly reduced the phosphorylation of estrogen receptors and the secretion of estradiol by ER^+^ breast cancer cells. CYP19A (aromatase) is an enzyme located in the endoplasmic reticulum, which plays a critical role in estradiol synthesis (aromatization), and we further demonstrated that ionizing radiation could induce endoplasmic reticulum stress with or without exogenous estradiol supplementation, and that it downregulated the expression of CYP19A through ER-phagy. In addition, ionizing radiation also promoted lysosomal degradation of CYP19A, reduced estradiol synthesis, and inhibited the proliferation of tamoxifen-resistant ER^+^ breast cancer cells. We concluded that ionizing radiation downregulated the expression of CYP19A and reduced estradiol synthesis by inducing endoplasmic reticulum stress in ER^+^ breast cancer cells, thereby ultimately inhibiting cellular proliferation.

## Introduction

According to the most recent data, breast cancer has officially replaced lung cancer as the most common cancer globally [1] and its most commonplace form is estrogen receptor-positive breast cancer [2]. Estrogens are important steroid hormones that play essential roles in cell growth and differentiation, and include estrone, estradiol, and estriol. Studies have shown that estradiol is principally produced in the adrenal glands, ovaries, placenta, testicles, adipose tissue, breast, and brain [3], and that its synthesis is catalyzed by the cytochrome P450 family of enzymes, of which aromatase CYP19A is particularly important [4, 5]. Women with high estradiol concentrations in their blood almost doubled their risk of breast cancer compared with those with low levels [6]. In vitro experiments have also revealed that estradiol stimulates the growth of breast cancer cells by activating estrogen receptor α (ERα) to upregulate the PI3K/Akt-signaling pathway [7]. Therefore, the regulation of estradiol synthesis is essential for the treatment of ERα^+^ breast cancer [3]. ERα is expressed in <10% of normal breast epithelium, but in about 50–80% of breast tumors [8]. Experimentally, the role of ERα in promoting tumorigenesis has been demonstrated in ERα knockout mice [9]. Although ERα antagonists exert the effect of abrogating estradiol synthesis, the development of drug resistance has become another immense challenge facing clinical practice. Therefore, the discovery of novel therapeutic methods with selective anti-estradiol potential remains an actively researched and exigent area of this field.

The endoplasmic reticulum (EnR) is an organelle responsible for regulating steroid synthesis. The EnR quality-control system selectively governs correctly folded proteins for transport and degrades misfolded proteins to maintain intracellular homeostasis. However, in some circumstances (such as with a lack of nutrition, hypoxia, or ionizing radiation), the EnR’s regulatory role becomes disturbed—causing accumulation of the misfolded or unfolded proteins—and this leads to EnR stress (ERS) [10]. In response to ERS, cells possess an integrated signaling system that includes the unfolded protein response (UPR) [11] and ER-associated degradation [12]. Under ERS, an unfolded protein binds to BIP (a chaperone protein), resulting in the separation of BIP from protein kinase R-like EnR kinase (PERK) and inositol-requiring enzyme 1 (IRE1) complex, and the activation of transcription factor 6 (ATF6), mediating the UPR [13]. In most mammals, UPR is mediated by IRE1α and XBP1 is spliced by IRE1α, and enters the nucleus to induce the transcription of UPR target genes, which in turn generates the upregulation of the EnR chaperone protein [14].

Autophagy is a catabolic process in which cytoplasmic material is transported to lysosomes for degradation and the EnR is the primary source of the formation of autophagosomes. Studies have shown that the EnR can also degrade its accumulation through selective autophagy, a process known as ER-phagy [15]. Lysosomes play an essential role in this degradation pathway and ERS can induce ER-phagy, which is manifested in the accumulation of LC3B-II and an increase in autophagosomes [16, 17]. The UPR also participates in the regulation of ER-phagy by primarily triggering the IRE1α/XBP1 pathway, thus promoting the transformation of LC3B-I into LC3B-II and the formation of autophagosomes [18]. In addition, the unfolded proteins stimulate the oligomerization of IRE1α on the EnR and autophosphorylation of IRE1α in the cytoplasmic domain, promoting the expression of XBP1 and mediating ER-phagy [19].

Endocrine therapy such as tamoxifen is commonly used in the clinical treatment of ER^+^ breast cancer, but it can also lead to drug resistance. Radiotherapy is an essential means of treating breast cancer, whether postoperative radiotherapy for early breast cancer or radiotherapy for local or distal metastases. As numerous investigators have demonstrated that ionizing radiation inhibits estrogen secretion in ER^+^ breast cancer cells [20, 21], we wished to explore whether it does so by reducing estradiol synthesis via ERS in ER^+^ breast cancer cells and thus inhibit cellular proliferation.

## Results

### Ionizing radiation inhibits the proliferation of breast cancer cells

**Fig. 1:**
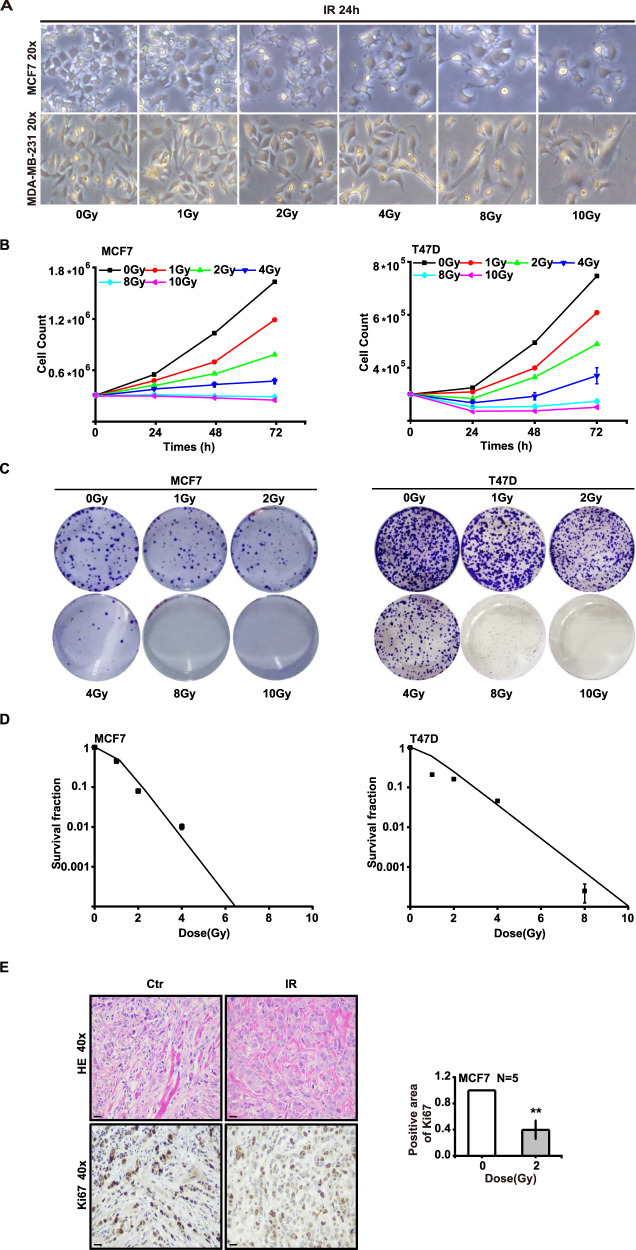
Ionizing radiation inhibits the proliferation of breast cancer cells. MCF7, T47D, and MDA-MB-231 cells were irradiated with X-rays at different doses. The morphology of MCF7 and MDA-MB-231 cells became swollen after irradiation at 24 h (**A**). Cellular proliferation (**B**) and clonal formation (**C**, **D**) of MCF7 and T47D cells were inhibited by radiation in dose- and time-dependent manners. Malignant proliferation decreased after 2 Gy of radiation in MCF7 tumor cells (**E**) (mean ± SD of triplicate assessments, Student’s *t*-test, **p < 0.01).

ER^+^ breast cancer cell MCF7 and T47D, and ER^−^ breast cancer cell MDA-MB-231 were irradiated with X-rays at different doses, and we found that 24 h after irradiation, MCF7 and MDA-MB-231 cells expansion and proliferation slowed commensurate with the increase in radiation dose (Fig. 1A). Furthermore, we used cell-counting and clone-formation assays to show that the proliferative inhibition of MCF7 and T47D cells were radiation dose-dependent and time-dependent (Fig. 1B). After large radiation doses of 8 and 10 Gy, the cells went into a state of nonproliferation, confirmed by the clone-formation experiment. On the 14th day (for MCF7 cells) and 21st day (for T47d cells), the clonal formation (Fig. 1C) and survival fraction (Fig. 1D) for the two types of cells decreased significantly, with virtually no clones observed after the largest doses of radiation. In addition, when we cultured MCF7 and T47D cells with a conditioned medium containing 10 nM estradiol, we noted that supplementation with exogenous estradiol significantly enhanced cellular proliferation and clonal formation (Supplementary Fig. S1A, B); we also demonstrated conversely that X-rays could inhibit cellular proliferation (Supplementary Fig. S1C) and clone formation (Supplementary Fig. S1D, E). In addition, we discerned that X-rays downregulated the expression of Ki67 in MCF7 tumors using immunohistochemistry (Fig. 1E). In conclusion, ionizing radiation inhibited the proliferation of breast cancer cells.

### Ionizing radiation downregulates phosphorylation of ERα

**Fig. 2:**
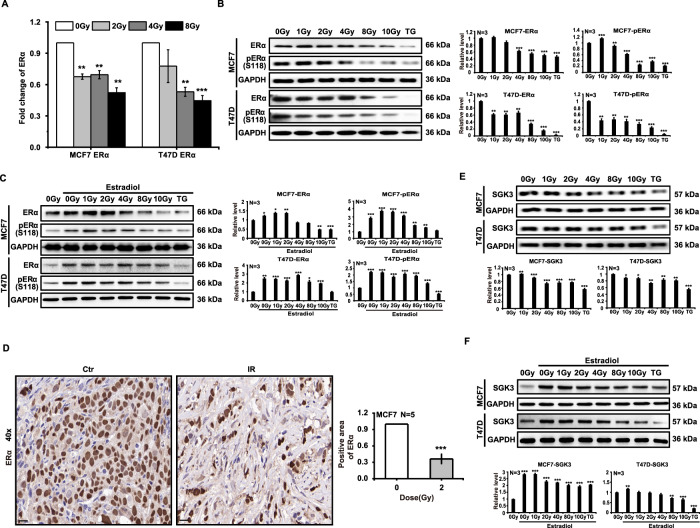
Ionizing radiation downregulates phosphorylation of ERα. MCF7 and T47D cells were irradiated with X-rays at different doses. Twenty-four hours after irradiation, ERα mRNA transcription decreased (**A**). Ionizing radiation downregulated the expression and phosphorylation of ERα without (**B**) or with (**C**) estradiol supplementation. The expression of ERα decreased after 2 Gy of radiation in MCF7 tumor cells (**D**). Ionizing radiation downregulated the expression of SGK3 without (**E**) or with (**F**) estradiol supplementation (mean ± SD of triplicate assessments, Student’s *t*-test, **p < 0.01, ***p < 0.001).

ERα is highly expressed in ER^+^ breast cancer cells and plays an important role in cellular proliferation. Our real-time quantitative PCR results showed that ERα mRNA expression declined with the X-ray irradiation of MCF7 and T47D cells (Fig. 2A). Wang et al. [22] reported that thapsigargin (TG) reduced the expression of ERα and we therefore chose TG as the positive control for our study. Western blotting results revealed that the expression of ERα in MCF7 cells was downregulated by 4 Gy of radiation and that ERα phosphorylation was also downregulated at large doses. ERα expression and ERα phosphorylation also diminished in T47D cells after irradiation with different doses of X-rays (Fig. 2B); identical results were found with estradiol supplementation (Fig. 2C), although exogenous estradiol can elevate ERα expression. In addition, we perceived that X-rays downregulated the expression of ERα in MCF7 tumors with immunohistochemistry (Fig. 2D). As serum glucocorticoid-regulated kinase 3 (SGK3) is a transcriptional target for ERα and promotes ER^+^ breast cancer cell survival [22], we irradiated MCF7 and T47D cells with different doses of X-rays and found that SGK3 expression declined (Fig. 2E). SGK3 expression was additionally upregulated in MCF7 and T47D cells when cultured with estradiol-conditioned medium and X-rays downregulated SGK3 expression as well (Fig. 2F). In conclusion, these results indicated that ionizing radiation reduced ERα expression and ERα phosphorylation in ER^+^ breast cancer cells.

### Ionizing radiation induces ER-phagy in ER^+^ breast cancer cells

**Fig. 3:**
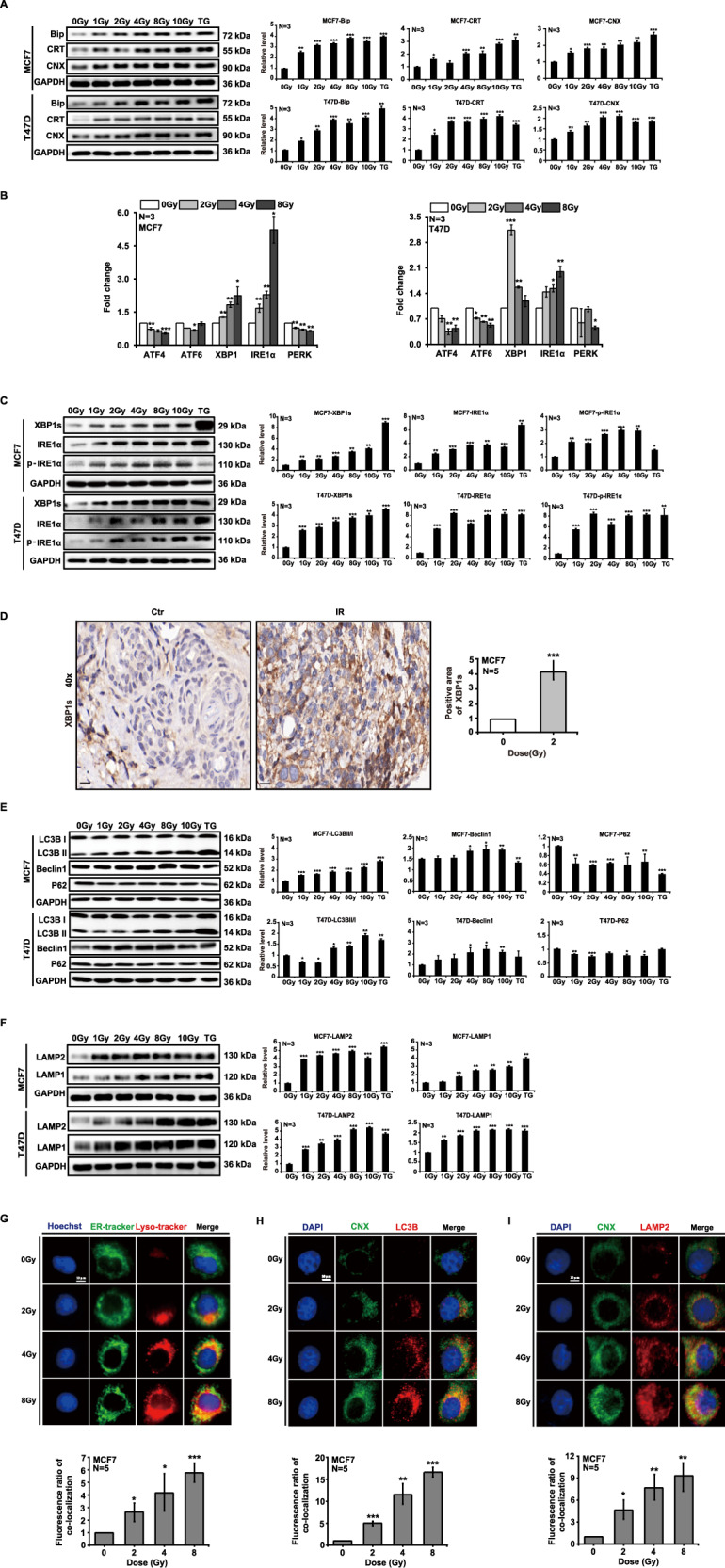
Ionizing radiation induces ER-phagy in ER^+^ breast cancer cells. MCF7 and T47D cells were irradiated with X-rays at different doses. Twenty-four hours after irradiation, the expression of endoplasmic reticulum stress marker proteins increased (**A**). Ionizing radiation upregulated the expression of XBP1s and IRE1α (**B**, **C**). The expression of XBP1s increased in MCF7 tumors after irradiation (**D**). The expression of the key autophagic proteins LC3B and Beclin1 increased, and P62 expression was attenuated (**E**). The expression of LAMP1 and LAMP2 was augmented (**F**). Irradiation induced morphologic shrinkage of the ENR and lysosomal aggregation increased (**G**). The colocalization of CNX/LC3B (**H**) and CNX/LAMP2 (**I**) increased with irradiation (mean ± SD of triplicate assessments, Student’s *t*-test, *p < 0.05, **p < 0.01, ***p < 0.001).

Ionizing radiation-induced ERS in tumor cells has been widely reported and in our study we focused on the ERS marker proteins BIP, CRT, and CNX. The expression of the above proteins was upregulated in MCF7 and T47D cells by X-ray irradiation at different doses (Fig. 3A). Similar protein expression was also found with exogenous estradiol supplementation (Supplementary Fig. S2A). ERS typically triggers the UPR mediated by the IRE1α/XBP1 pathway. We thus further examined gene expression changes and found that IRE1α and XBP1 were significantly upregulated in MCF7 and T47D cells after irradiation (Fig. 3B). Western blotting analysis also showed that the expression of IRE1α, XBP1s, and the phosphorylation of IRE1α increased in a dose-dependent manner in MCF7 and T47D cells after irradiation (Fig. 3C); the expression changes were similar in the presence of exogenous estradiol (Supplementary Fig. S2B). X-rays also upregulated the expression of XBP1s in MCF7 tumors upon immunohistochemical evaluation (Fig. 3D). These results suggested that ionizing radiation induced ERS by activating the IRE1α/XBP1-signaling axis in ER^+^ breast cancer cells. We also observed that the autophagic marker protein LC3B-II/I ratio was increased, that Beclin1 expression was upregulated, and that the expression of P62 was downregulated by western blotting analysis in MCF7 and T47D cells after X-ray irradiation (Fig. 3E). We likewise detected autophagy in the exogenous estradiol-supplemented condition (Supplementary Fig. S2C) and the expression of the lysosomal marker proteins LAMP1 and LAMP2 was augmented in the presence (Supplementary Fig. S2D) or absence of exogenous estradiol (Fig. 3F). When we further examined whether ionizing radiation caused ER-phagy, we noted by ER-tracker and immunofluorescence staining that X-rays caused shrinkage of EnR morphology, and that lysosomal aggregation increased (Fig. 3G). The colocalization of CNX/LC3B (Fig. 3H) and CNX/LAMP2 (Fig. 3I) increased with irradiation. These results suggested that ionizing radiation induced ER-phagy mediated by activation of the IRE1α/XBP1 pathway in ER^+^ breast cancer cells.

### Ionizing radiation downregulates the expression of CYP19A

**Fig. 4:**
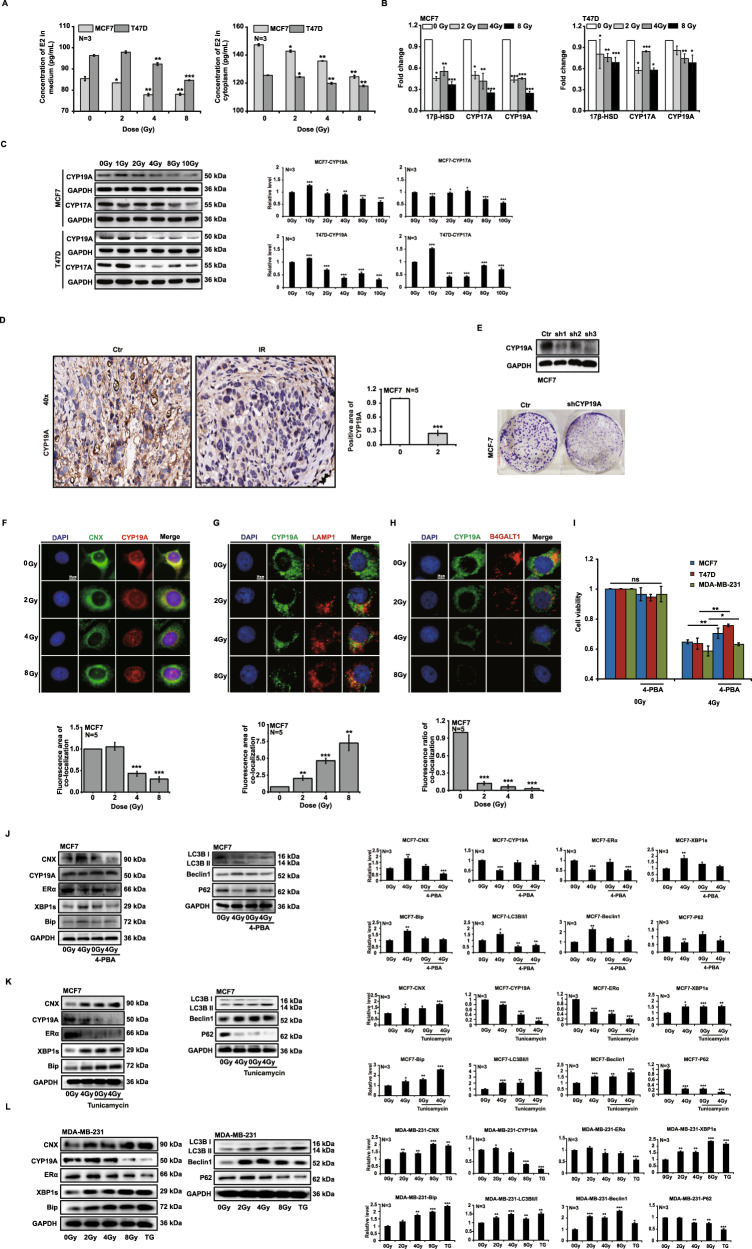
Ionizing radiation downregulates the expression of CYP19A. MCF7 and T47D cells were irradiated with X-rays at different doses. Twenty-four hours after irradiation, the secretion of estradiol decreased (**A**). Irradiation downregulated CYP19A expression in vivo and in vitro (**B**–**D**). CYP19A^−/−^ MCF7 cell clonal formation was significantly slowed (**E**). Irradiation reduced the colocalization of CNX and CYP19A (**F**), and increased the colocalization of CYP19A and lysosomes (**G**). Irradiation inhibited CYP19A transport from the EnR to the Golgi complex (**H**). 4-PBA rescued ionizing radiation-induced cell death (**I**). In the case of endoplasmic reticulum stress (ERS) inhibition, the expression of CYP19A and ERα was not significantly decreased after irradiation and autophagy was also inhibited (**J**). The expression of CYP19A declined by ionizing radiation and ERS inducer co-treatment (**K**). Ionizing radiation induced ERS and autophagy in ER^−^ breast cancer cell (**L**) (mean ± SD of triplicate assessments, Student’s *t*-test, *p < 0.05, **p < 0.01, ***p < 0.001).

We uncovered a significant reduction in estradiol secretion by ER^+^ breast cancer cells (Fig. 4A), ER^−^ breast cancer cells (Supplementary Fig. S3A), and normal breast cells (Supplementary Fig. S3B) upon ionizing radiation, and thus used real-time quantitative PCR assay to determine the mRNA expression for 17β-HSD, CYP17A, and CYP19A enzymes of the estradiol synthetic pathway. The results showed that CYP19A mRNA expression decreased in MCF7 and T47D cells after irradiation (Fig. 4B), and we noted that CYP19A protein expression was also reduced (Fig. 4C). We subsequently found CYP19A to be significantly upregulated under treatment with the estradiol-conditioned medium in MCF7 and T47D cells (Supplementary Fig. S3C), and downregulated by X-ray irradiation using different doses (Supplementary Fig. S3D). Ultimately, we showed by immunohistochemistry that X-rays downregulated the expression of CYP19A in MCF7 tumors (Fig. 4D). We used lentivirus shRNA to knock out CYP19A in MCF7 cells and demonstrated that CYP19A^−/−^ MCF7 cell clone formation was significantly slowed (Fig. 4E). As CYP19A is synthesized in the EnR, radiation should cause ERS and lead to ER-phagy. Therefore, we chose to explore the status of CYP19A in the EnR after irradiation. According to our immunofluorescence results, we confirmed that CYP19A was located in the EnR, and that irradiation reduced the colocalization of CNX and CYP19A (Fig. 4F). Next, we found the number of lysosomes to be increased, and that the colocalization of CYP19A and lysosomes increased after different doses of X-ray radiation in MCF7 cells (Fig. 4G); this phenomenon also took place with exogenous estradiol supplementation (Supplementary Fig. S3E). Colocalization of CYP19A and Golgi bodies was significantly reduced after irradiation, indicating that CYP19A was no longer transported from the EnR to the Golgi (Fig. 4H). In addition, we found that ERS inhibitor 4-PBA (4-Phenylbutyric acid) rescued the cell death induced by ionizing radiation in ER^+^ breast cancer cells but did not significantly affect ER^−^ breast cancer cells (Fig. 4I). Although we inhibited ERS by using 4-BPA, the expression of CNX, XBP1, and BIP in MCF7 cells did not change significantly after irradiation. The decreasing trends shown for CYP19A and ERα were not obvious, and autophagy was also inhibited (Fig. 4J). Furthermore, we found that ERS inducer Tunicamycin significantly improved the autophagy in MCF7 cells and slightly downregulated the expression of CYP19A. When MCF7 cells were co-treated with ionizing radiation and Tunicamycin, CYP19A expression was remarkably decreased (Fig. 4K). In addition, we also found that ionizing radiation could induce ERS and autophagy in MDA-MB-231 cells, but the expression of CYP19A was declined only at large doses (Fig. 4L). In conclusion, these results indicated that ionizing radiation downregulated the expression of CYP19A via ERS.

### Ionizing radiation inhibits the proliferation of MCF7/TAM cells

**Fig. 5:**
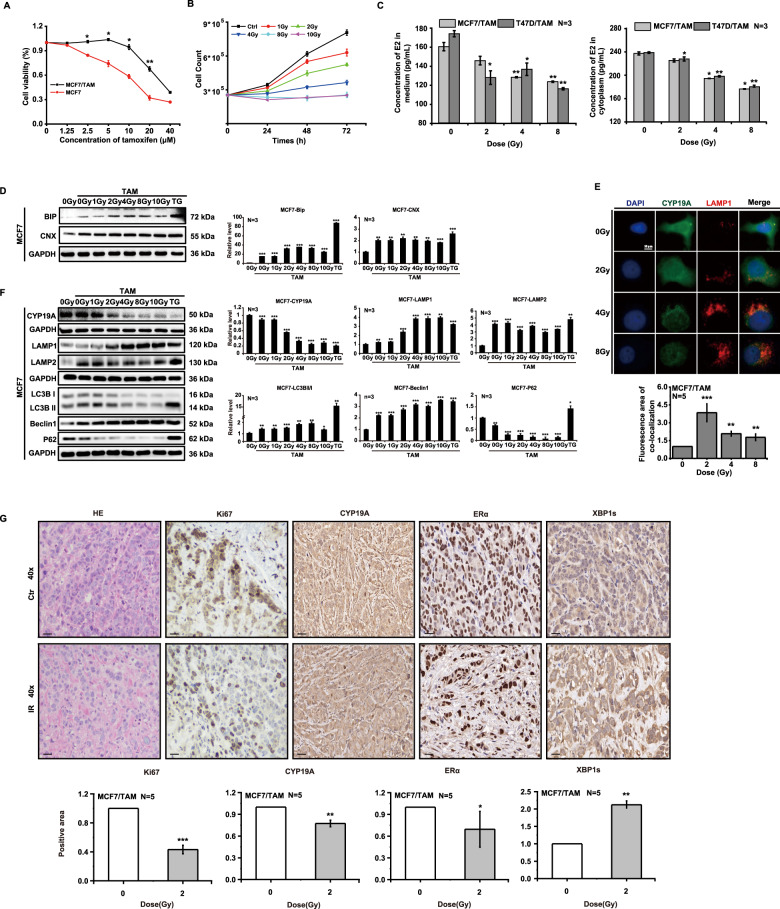
Ionizing radiation inhibits the proliferation of MCF7/TAM cells. MCF7/TAM and MCF7 cells were treated with 4-OH TAM at different doses for 24 h. The proliferation of MCF7/TAM was normal at 10 μM, whereas that of MCF7 was significantly inhibited (**A**). Irradiation inhibited the proliferation of MCF7/TAM cells in dose- and time-dependent manners (**B**), and diminished estradiol secretion (**C**). The expression of BIP and CNX proteins was upregulated after irradiation (**D**). Irradiation induced the colocalization of CYP19A and lysosomes increased in number (**E**). Ionizing radiation downregulated the expression of CYP19A and increased LAMP1 and LAMP2 expression; the expression of LC3B and Beclin1 increased and that of P62 decreased (**F**). Irradiation downregulated the expression of Ki67, CYP19A, ERα, and upregulated the expression of XBP1s in MCF7/TAM tumors (**G**) (mean ± SD of triplicate assessments, Student’s *t*-test, *p < 0.05, **p < 0.01, ***p < 0.001).

In order to explore the inhibitory mechanism(s) governing ionizing radiation effects on MCF7/TAM cells, we used CCK8 kits to detect cellular proliferation. The results showed that MCF7/TAM cells grew well at a maximal concentration of 10 μM 4-hydroxytamoxifen (4-OH TAM) treatment over 24 h (Fig. 5A); when irradiated with X-rays, their cell counts showed that ionizing radiation inhibited their proliferation in dose- and time-dependent manners (Fig. 5B), and that their secretion of estradiol decreased (Fig. 5C). Wef also found that the expression of BIP and CNX proteins was upregulated after irradiation in MCF7/TAM cells (Fig. 5D). Immunofluorescence revealed that colocalization of CYP19A and LAMP1 increased after irradiation (Fig. 5E), and western blot analysis showed a diminution in CYP19A expression and an elevation in LAMP expression. When we evaluated the expression of autophagic marker proteins in MCF7/TAM cells, we observed that the LC3B-II/I ratio and Beclin1 expression were augmented with irradiation, especially after large doses of X-rays, and that P62 decreased in a dose-dependent manner (Fig. 5F). Finally, we depicted in tumors a downregulation by X-rays in the expression of Ki67, CYP19A, and ERα, and an upregulation in the expression of XBP1s (Fig. 5G). These results showed that MCF7/TAM cells experienced ERS and induced ER-phagy at a large X-ray dose, resulting in the downregulation of CYP19A expression and inhibition of cellular proliferation.

## Discussion and conclusions

Steroidogenic enzymes play an essential role in the regulation of estradiol production and one of these enzymes, CYP19A, was our focus in the current study. Alonso-Gonzalez et al. [23] found that ionizing radiation inhibited aromatase expression, thereby inhibiting estrogen synthesis in ER^+^ breast cancer cells, and this is consistent with our findings. Except for the occasional upregulation of CYP19A expression after 1 Gy of radiation, the other doses significantly reduced the expression of CYP19A (the 1 Gy dose might have induced a stress response). MCF7 and T47D cells cultured in estradiol-conditioned medium promoted the expression of CYP19A and further synthesized more estradiol. Ionizing radiation also significantly inhibited the expression of CYP19A in ER^+^ breast cancer cells cultured in medium supplemented with exogenous estradiol, indicating that the inhibition of breast cancer cell proliferation by ionizing radiation was not affected by exogenous estradiol. In addition, ionizing radiation can also reduce the secretion of estradiol in ER^−^ breast cancer cells and normal breast cells but was more sensitive to ER^+^ breast cancer cells.

The elevated expression of ERα is the most significant characteristic of ER^+^ breast cancer. ERα typically dimerizes when bound with estradiol and acts as a ligand-inducible transcription factor in the nucleus so as to occupy a regulatory role. Although MCF7 and T47D cell lines showed differential sensitivities to radiation, ionizing radiation reduced the phosphorylation of ERα with or without exogenous estradiol supplementation. In addition, estradiol has been reported to induce SGK3 expression in dose- and time-dependent manners by binding with ERα to promote cellular survival and proliferation, and in our study, irradiation significantly inhibited the overall expression of SGK3.

It is indisputable that ionizing radiation causes ERS. Kim et al. [24] reported that 15 Gy of X-ray radiation selectively activated the eIF2α/ATF4-signaling pathway in human umbilical vein endothelial cells. In our study, ionizing radiation upregulated the expression of BIP, CRT, and CNX, inducing ERS. As we observed an increase in the lysosomal number, we hypothesized that ERS would lead to ER-phagy. By analyzing the UPR effector proteins PERK, ATF6, and IRE1, we found that IRE1α/XBP1 mediated the ER-phagy induced by ionizing irradiation of MCF7 and T47D cells. Zhang et al. [25] showed that the overexpression of XBP1s in liver cells enhanced transcription factor EB transcription and led to autophagy, and Chaurasia et al. [26] found that ionizing radiation caused autophagy in macrophages and promoted proliferation via IRE1 and PERK. In our study, we established that the expression of IRE1α and XBP1s was significantly upregulated in ER^+^ breast cancer cells after irradiation. LC3B and CNX were additionally fully colocalized and the expression of LC3B and Beclin1 were upregulated considerably after irradiation, leading to the downregulation of P62 and indicating that ionizing radiation promoted autophagy in ER^+^ breast cancer cells. CYP19A was localized as expected to the EnR and the degradative process with respect to CYP19A was revealed by the colocalization of CNX and CYP19A, ER-tracker and LYSO-tracker, and CYP19A and LAMP1.

Consistent with previous reports, ionizing radiation can cause severe EnR “damage” to drug-resistant cell lines. After irradiation, MCF7/TAM and T47D/TAM showed different morphologies relative to normal cells, i.e., they became enlarged and grew more slowly with the increasing radiation dose. Although CYP19A was affected differentially in MCF7/TAM and T47D/TAM cell lines, irradiation nevertheless promoted CYP19A degradation through ER-phagy and reduced the synthesis of estradiol. IRE1α also mediated UPR in tamoxifen-resistant cell lines, the difference being that IRE1α and XBP1s of irradiated tamoxifen-resistant cells principally increased after large doses of radiation. Interestingly, after irradiation, LC3B in MCF7/TAM and T47D/TAM cells was also differentially expressed: in the former cell line, LC3B was only observed after high-dose irradiation, whereas in the latter cell line the effect was not obvious, which may be due to a particular phenomenon with respect to the cell type. We also uncovered a similar situation in T47D cells cultured with or without estradiol. Although the expression of P62 was significantly downregulated in both cell lines, the alterations in Beclin1 in MCF7/TAM cells were only observed at high-dose irradiation; in contrast, Beclin1 in T47D/TAM was increased dose-dependently. ER-phagy was not identical between the two tamoxifen-resistant cell lines, which we hypothesized to be caused by genetic reprogramming.

Therefore, we concluded that ionizing radiation downregulated the phosphorylation of ERα and diminished the secretion of estradiol by inducing ERS and inhibiting CYP19A synthesis, thereby attenuating the proliferation of ER^+^ breast cancer cells.

## Materials and methods

### Radiotherapy

X-rays were generated with an X-Rad 225 generator (energy level, 225 kV/13.3 mA; Precision X-ray (PXi), North Branford, USA). The cells or breast tumor-bearing nude mice were placed on a platform after anesthesia and the mice were protected by a lead plate, with only the tumor site exposed to radiation.

### Cell lines

We purchased the ER^+^ breast cancer cell lines MCF7 and T47D, ER^−^ breast cancer cell line MDA-MB-231, and ER^+^ breast cell line MCF10A from the Chinese Academy of Sciences Cell Bank. MCF7, T47D, and MDA-MB-231 were cultured in Dulbecco’s modified Eagle’s medium (DMEM) containing 10% fetal bovine serum (FBS), 100 U/mL of penicillin, and 100 μg/mL of streptomycin. MCF10A was cultured in DMEM containing 5% horse serum, 20 ng/mL epidermal growth factor, 0.5 μg/mL Hydrocortisone, 10 μg/mL Insulin, 1% Non-Essential Amino Acids (NEAA), 100 U/mL of penicillin, and 100 μg/mL of streptomycin. Culture conditions were maintained at 37 °C and 5% CO_2_ in compressed air in a constant-temperature incubator with saturated humidity.

#### Estradiol-conditioned medium cell culture

When the cells grew to 50% confluency in a 35 mm culture dish, they were washed with sterile phosphate buffered saline (PBS) and replaced with phenol red-free and serum-free DMEM for 24 h of incubation. We then cultured the cells in DMEM containing 10 nM estradiol and 10% FBS. Culture conditions were maintained at 37 °C and 5% CO_2_ in compressed air in a constant-temperature incubator with saturated humidity.

#### Construction of drug-resistant cell lines

Construction of drug-resistant cell lines: MCF7 and T47D cells were grown to 70% confluency in 35 mm culture dishes containing DMEM and we replaced the medium with 1 μM 4-OH TAM-supplemented medium for 48 h. After removal of the conditioned medium, the cells were cultured in a typical manner. We repeated the aforementioned procedures for almost 6 months until the cells grew steadily in the medium containing 4-OH TAM at a final concentration of 10 μm for MCF7 cells and at a final concentration of 2 μM for T47D cells.

### Experimental mouse model

Six-week-old BALB/C nude mice were purchased from Xi’an Keao Biological Technology Co., Ltd and maintained in the animal facility at the Institute of Modern Physics in specific pathogen-free conditions and a temperature-controlled environment with 12 h light, 12 h dark cycles. Animals received food and water ad libitum. All of the animal experiments were approved by the Ethical Committee of the Institute of Modern Physics and followed EU Directive 2010/63/EU guidelines. MCF7 and MCF7/TAM tumors were established in BALB/C nude mice hosts by subcutaneously inoculating 1,000,000 cells per mouse. When the tumors became palpable (at 50 mm^3^), they were locally irradiated with 2 Gy doses of X-rays. The MCF7/TAM group was intraperitoneally injected with TAM (0.01 g/20 g BW) three times per week.

### Western immunoblotting analysis

Cells were collected into RIPA lysate (150 mM sodium chloride, 1.0% NP-40, 0.5% sodium deoxycholate, 0.1% SDS, and protease inhibitor cocktails), ultrasonicated, and then mixed in 5× loading buffer. The protein was placed in a 98 °C metal bath and heated for 10 min for denaturation. We used constant-pressure electrophoresis, with the voltage set at 80 V for protein concentration and 120 V for separation. The membrane was transferred at 120 V and 0.2 A—with the specific transfer time depending upon the molecular weight—and incubated with 5% bovine serum albumin at room temperature for 2 h and with the primary antibody overnight at 4 °C. The next day, after washing five times with Phosphate Buffered Solution plus Tween-20 (PBST), a horseradish peroxidase-labeled secondary antibody was added and the membrane was incubated at room temperature for 90 min. Images were exposed by electro-chemiluminescence immunoassay and analyzed with Image J software. All of the information regarding antibodies is shown in Supplementary Table 1.

### Real-time quantitative PCR

We used the Trizol method to extract RNA and cDNA was obtained by taking 3 μg of RNA for reverse transcription according to the GeneCopopoeia reverse-transcription kit manual. The cDNA was diluted three times and we utilized the SYBR Green system for real-time quantitative detection. The final volume was 20 μL and we executed a total of 40 cycles. Pre-denaturation was implemented at 95 °C for 10 min, denaturation at 95 °C for 10 s, annealing at 60 °C for 20 s, and primer template extension at 72 °C for 15 s—under a heating rate of 0.5 °C/6 s between 72 °C and 95 °C. All of the data collection was completed using the Bio-Rad CFX96 PCR system software. The collected Ct values were calibrated with glyceraldehyde 3-phosphate dehydrogenase as an internal reference and the target gene expression was analyzed using the ^2-∆^CT method. Primers and sequences are shown in Supplementary Table 2.

### Immunofluorescence

#### Live-image collection

Cells were incubated in medium mixed with Lyso-Tracker Red (60 nM) and ER-Tracker™ Green (1 μm) for 10 min in the dark. Then Hoechst 33342 (1 μm) was added and the cells continued to incubate at room temperature for 10 min. We collected the images under fluorescence microscopy (RVL-100-G, ECHO, California, USA) .

#### Fixed image collection

Paraformaldehyde (4%) was used to fix the cells for ~10 min. We then discarded the supernatant, incubated the cells with cold methanol for 20 min, and ultimately prepared the cells by cleaning them with 75% glacial ethanol. After rehydration, 0.5% Triton X-100 was used to permeabilize the cell membrane for ~5 min. The samples were placed in goat serum diluted with PBS (1 : 20) and blocked for 2 h, followed by the addition of primary antibody (dilution ratio, 1 : 500) and incubation for 2 h. After rinsing five times with PBST, the corresponding fluorescent dye-conjugated secondary antibody (dilution ratio, 1 : 1000) was added and the cells were incubated in the dark for 1.5 h. We then added 10 μl of 4′,6-diamidino-2-phenylindole and collected the images under fluorescence microscopy. All of the information regarding chemical products are shown in Supplementary Table 3.

### Immunohistochemistry

The tumors were embedded in paraffin and sectioned at 3 μm. After dewaxing and hydration, the slices were placed in 0.01 M sodium citrate buffer solution (pH 6.0) and heated at 95 °C for 10 min. We then used 0.5% Triton X-100 to permeabilize the cell membrane and 3% H_2_O_2_ to remove the endogenous peroxides. After blocking, we incubated the cells with primary antibodies against Ki67 (1 : 500), ERα (1 : 500), XBP1 (1 : 200), and CYP19A (1 : 200) at 4 °C overnight. On the second day and after PBST washing, biotin was added to label the secondary antibodies. The antibodies were incubated at room temperature for 30 min and the cells then underwent DAB (3,3'-diaminobenzidine) staining and hematoxylin red dyeing.

### Enzyme-linked immunosorbent assay

The quantification of estradiol was performed by means of an enzyme-linked immunosorbent assay Human E2 ELISA kit (#ml1077398-J, Shanghai Enzyme-linked Biotechnology Co., Ltd, Shanghai, China), following the manufacturer’s instructions. Absorbance was analyzed by means of an i3 Paradigm multi-label reader (Molecular Devices). We found that FBS had no interference with the results of estradiol secretion. Therefore, we did not specifically choose charcoal-stripped serum to complete this experiment.

### Statistical analysis

Origin 9.0 was used to analyze the results and the data are presented as *x* ± SE. We used Student’s *t*-test to evaluate significant differences and *P < 0.05, **P < 0.01, and ***P < 0.001 were considered to be statistically significant. Each experiment was performed independently at least three times.

## Supplementary information

Supplementary figure legends

Supplementary Figure 1

Supplementary Figure 2

Supplementary Figure 3

Supplementary table 1

Supplementary table 2

Supplementary table 3

## Data Availability

All data generated and analyzed during this study are included in this article. Each experiment was performed at least three times independently.
