# Supplementary material for: Ionizing radiation downregulates estradiol synthesis via endoplasmic reticulum stress and inhibits the proliferation of estrogen receptor-positive breast cancer cells
Source: Cell Death Dis. 2021 Oct 29;12(11):1029. doi: 10.1038/s41419-021-04328-w (PMC8556230; doi:10.1038/s41419-021-04328-w)
Supplement: Supplementary file 1 — Supplementary figure legends [file 41419_2021_4328_MOESM1_ESM.docx]

**Supplementary Figures**

**S1.** **Ionizing radiation inhibits the proliferation of estradiol-stimulated ER^+^ breast cancer cells.**

MCF7 and T47D cells were cultured in 10 nM estradiol-supplemented medium. Exogenous estradiol enhanced cellular proliferation and clonal formation (A, B). Irradiation inhibited cellular proliferation (C) and clonal formation (D,E) in dose- and time-dependent manners.

**S2. Ionizing radiation induces ER-phagy** **in estradiol-stimulated ER^+^ breast cancer cells.**

MCF7 and T47D cells were cultured in 10 nM estradiol-supplemented medium. Irradiation up-regulated the expression of Bip, CRT, CNX (A), XBP1s and IRE1α (B); expression of LC3B and Beclin1 was increased while that of P62 was reduced (C); and expression of LAMP1 and LAMP2 was slightly increased (D).

**S3. Ionizing radiation down-regulates the expression of CYP19A in estradiol-stimulated ER^+^ breast cancer cells.**

Ionizing radiation down-regulated the estradiol secretion in MDA-MB-231 and MCF10A cells (A,B). Exogenous estradiol up-regulated the expression of CYP19A (C). MCF7 and T47D cells were cultured in 10 nM estradiol-supplemented medium. Ionizing radiation down-regulated CYP19A expression (D). Co-localization of CYP19A and lysosomes increased after irradiation (E). (mean ± SD of triplicate assessments, Student’s *t* test, **p<0.01, ***p<0.001)
