# Supplementary figures and images for: Ionizing radiation downregulates estradiol synthesis via endoplasmic reticulum stress and inhibits the proliferation of estrogen receptor-positive breast cancer cells
Source: Cell Death Dis. 2021 Oct 29;12(11):1029. doi: 10.1038/s41419-021-04328-w (PMC8556230; doi:10.1038/s41419-021-04328-w)

Figure S1

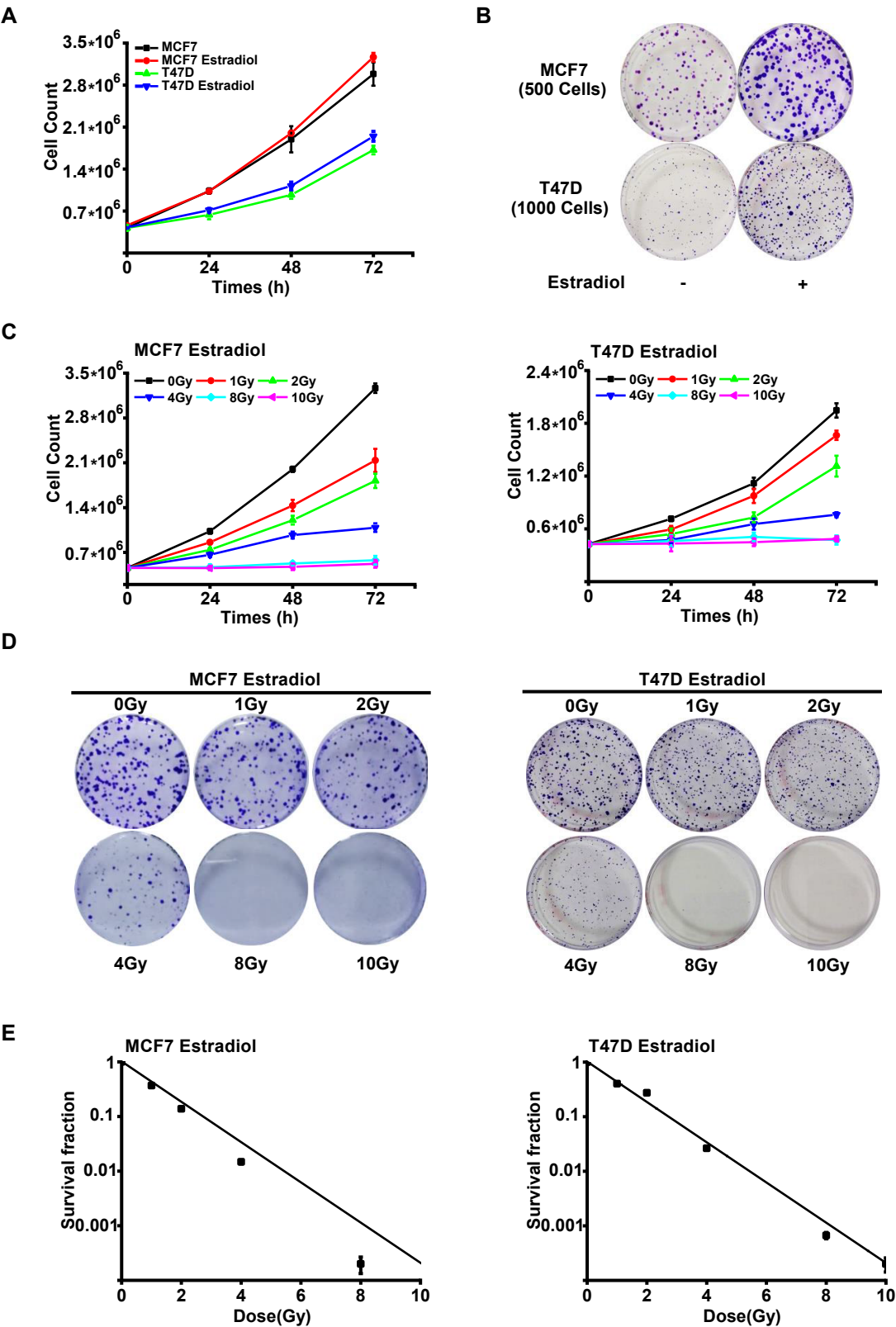

Supplement: Supplementary file 2 — Supplementary Figure 1 [file 41419_2021_4328_MOESM2_ESM.pdf]

Figure S2

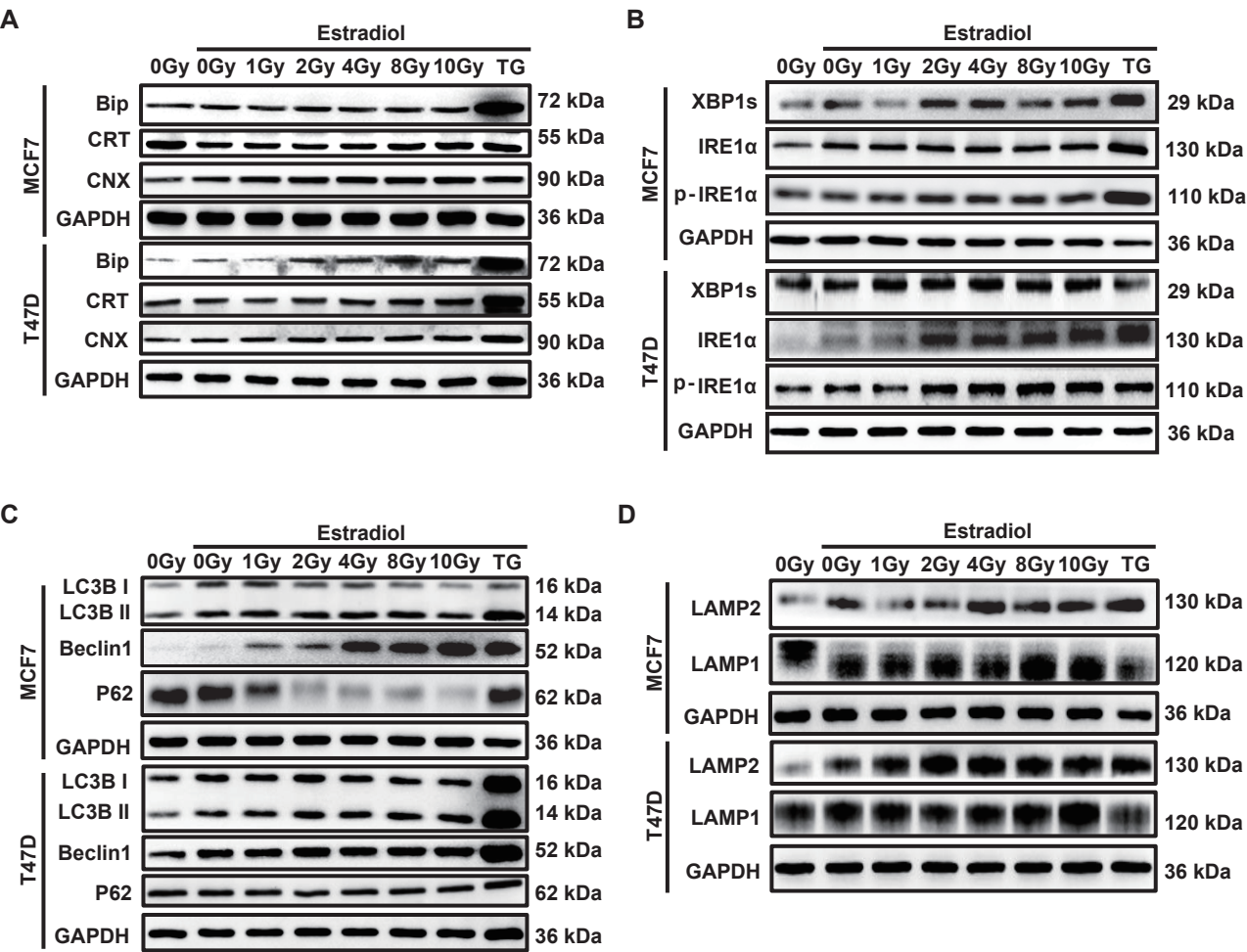

Supplement: Supplementary file 3 — Supplementary Figure 2 [file 41419_2021_4328_MOESM3_ESM.pdf]

Figure S3

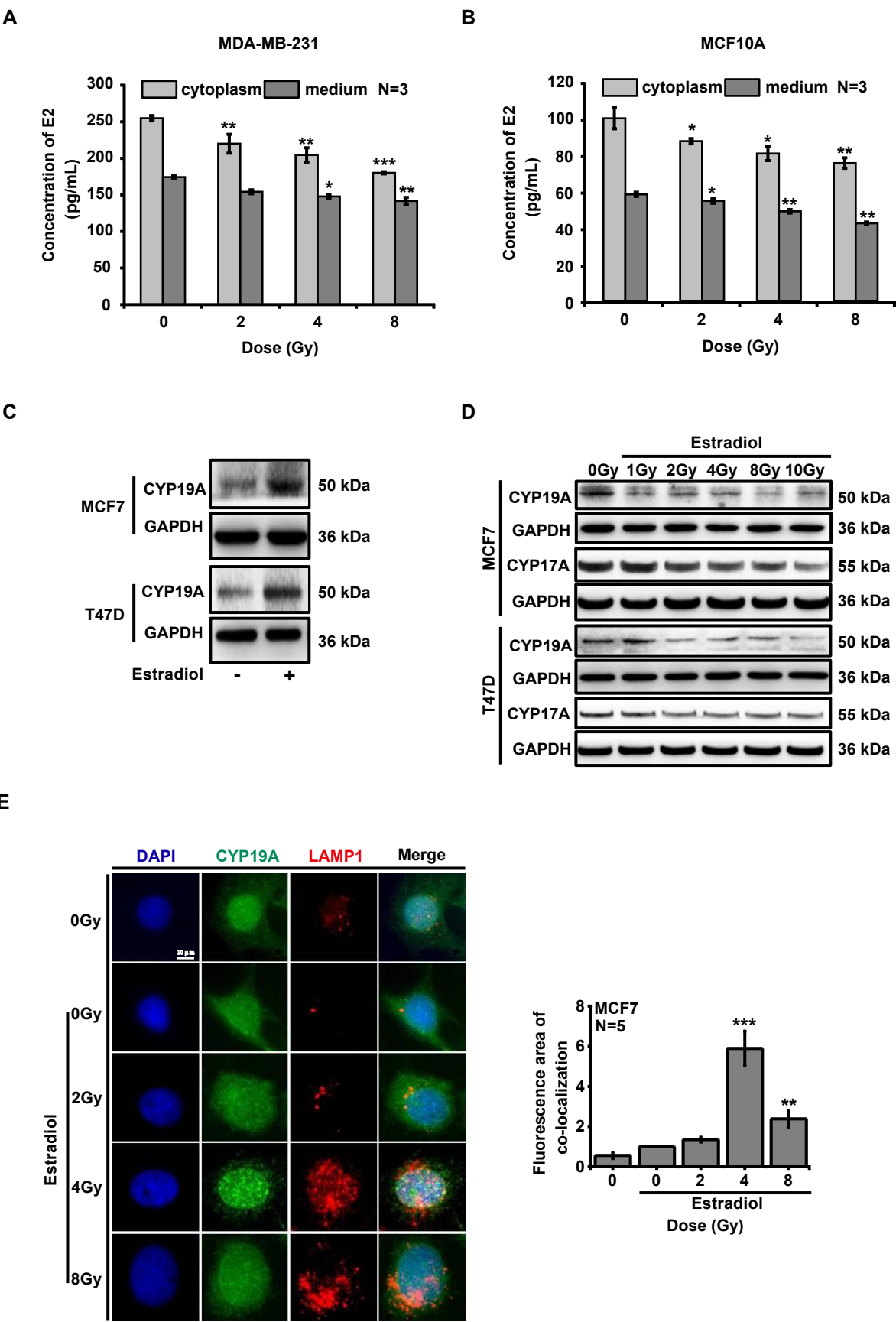

Supplement: Supplementary file 4 — Supplementary Figure 3 [file 41419_2021_4328_MOESM4_ESM.pdf]
