# Supplementary material for: Ionizing radiation downregulates estradiol synthesis via endoplasmic reticulum stress and inhibits the proliferation of estrogen receptor-positive breast cancer cells
Source: Cell Death Dis. 2021 Oct 29;12(11):1029. doi: 10.1038/s41419-021-04328-w (PMC8556230; doi:10.1038/s41419-021-04328-w)
Supplement: Supplementary file 5 — Supplementary table 1 [file 41419_2021_4328_MOESM5_ESM.docx]

**Table 1. List of antibodies**

| **Antibodies** | **Source** | **Identifier** |
| --- | --- | --- |
| ERα | CST | D8H8 |
| p-ERα（S118） | Santa cruz | sc-12915 |
| SGK3 | Protein tech | 12699-1-AP |
| CYP19A | Santa cruz | [sc-374176](https://www.scbt.com/zh/p/cyp19-antibody-e-9?requestFrom=search) |
| CYP19A HRP | Santa cruz | sc-374176 HRP |
| CYP17A | Santa cruz | [sc-374244](https://www.scbt.com/zh/p/cyp17a1-antibody-d-12?requestFrom=search) |
| Bip | Abcam | Ab108615 |
| Calreticulin | CST | D3E6 |
| Calnexin | Proteintech | 66903-1-1g |
| XBP1s | Proteintech | 24868-1-AP |
| XBP1s | Abcam | ab220783 |
| LAMP1 | CST | D401S |
| LAMP2 | Abcam | ab199946 |
| LAMP2 | Proteintech | 27823-1-AP |
| LC3B | Abcam | ab192890 |
| LC3B | Proteintech | 14600-1-AP |
| P62 | Abcam | ab207612 |
| p-IRE1α | Abcam | ab124945 |
| Beclin1 | Abcam | ab207612 |
| IRE1α | CST | 14C10 |
| B4GALT1 | Abcam | ab121326 |
