# Supplementary material for: Ionizing radiation downregulates estradiol synthesis via endoplasmic reticulum stress and inhibits the proliferation of estrogen receptor-positive breast cancer cells
Source: Cell Death Dis. 2021 Oct 29;12(11):1029. doi: 10.1038/s41419-021-04328-w (PMC8556230; doi:10.1038/s41419-021-04328-w)
Supplement: Supplementary file 6 — Supplementary table 2 [file 41419_2021_4328_MOESM6_ESM.docx]

**Table 2. List of primers**

| **Name** | **Sequence** |
| --- | --- |
| ERα | F: TACTGCATCAGATCCAAGGGAA  R: CCTCGGGGTAGTTGTACAC |
| CYP19A | F:GTGCTATTTGTCATCTGCTCCTGT  R:CATTTGGTGGAATCGGGTCT |
| CYP17A | F:ACARCTCCTTTCCTGTCTTCGTG  R:GGCTGTCTTTGCTCAGGTTGT |
| 17β-HSD | F:ACTAGTCGTGCAACGTCTACAGCAA  R:GCTCCCACTCCAAGGGCATA |
| ATF4 | F: ATGGATTTGAAGGAGTTCGACT  R: AGAGATCACAA GTGTCATCCAA |
| ATF6 | F: CTGATGGCTGTTCAATACACAG  R: GATCCCTTCGAAATGACACAAC |
| XBP1 | F: CTTGTAGTTGAGAACCAGGAGT  R: CCCAACAGGATATCAGACTCTG |
| IRE1α | F: CGTGAGCGACAGAATAGAAAAG  R: GCTTCTTATTTCTCATGGCTCG |
| PERK | F: CCAGTTTTGTACTCCAATTGCA  R: CAGATACAGCTGGCCTCTATAC |
| GAPDH | F:GCACCGTCAAGGCTGAGAAC  R:TGGTGAAGACGCCAGTGGA |
