# Supplementary material for: Ionizing radiation downregulates estradiol synthesis via endoplasmic reticulum stress and inhibits the proliferation of estrogen receptor-positive breast cancer cells
Source: Cell Death Dis. 2021 Oct 29;12(11):1029. doi: 10.1038/s41419-021-04328-w (PMC8556230; doi:10.1038/s41419-021-04328-w)
Supplement: Supplementary file 7 — Supplementary table 3 [file 41419_2021_4328_MOESM7_ESM.docx]

**Table 3 List of chemical products**

| **Reagents** | **Sources** | **Identifier** |
| --- | --- | --- |
| 17β-estradiol | Sigma | 50-28-2 |
| 4-hydroxytamoxifen | Sigma | 68392-35-8 |
| Fotel Bovine Serum | BI | 04-201-1B |
| Puromycin Dihydrochloride | MCE | 58-58-2 |
| Thapsigargin | MCE | 67526-95-8 |
| CYP19 shRNA | Santa cruz | sc-41498-V |
| 4-Phenylbutyric acid | MCE | 1821-12-1 |
| Tunicamycin | MCE | 11089-65-9 |
| Mito-Tracker Green | Invitrogen | 1863909 |
| Lyso-Tracker Red | Beyotime | C1046 |
| Hoechst 33342 | Invitrogen | 31661W |
| Radio Immunoprecipitation Assay Lysis buffer | Beyotime | P0013C |
| TRIpure Reagen | Roche | 11667165001 |
| PhosSTOP | Roche | 04906837001 |
| Protease Inhibitor Mix | Roche | 04693116001 |
